# Planning Steps Forward in Development: In Girls Earlier than in Boys

**DOI:** 10.1371/journal.pone.0080772

**Published:** 2013-11-27

**Authors:** Josef M. Unterrainer, Nina Ruh, Sandra V. Loosli, Katharina Heinze, Benjamin Rahm, Christoph P. Kaller

**Affiliations:** 1 Medical Psychology and Medical Sociology, University Medical Center Mainz, Mainz, Germany; 2 Department of Neurology, University Medical Center, University of Freiburg, Freiburg, Germany; 3 Freiburg Brain Imaging Center, University of Freiburg, Freiburg, Germany; 4 Cochlear Implant Center Erlangen, Ear, Nose and Throat (ENT) Department, University Hospital Erlangen, Erlangen, Germany; Birkbeck, University of London, United Kingdom

## Abstract

The development of planning ability in children initially aged four and five was examined longitudinally with a retest-interval of 12 months using the Tower of London task. As expected, problems to solve straightforward without mental look-ahead were mastered by most, even the youngest children. Problems demanding look-ahead were more difficult and accuracy improved significantly with age and over time. This development was strongly moderated by sex: In contrast to coeval boys, four year old girls showed an impressive performance enhancement at age five, reaching the performance of six year olds, whereas four year old boys lagged behind and caught up with girls at the age of six, the typical age of school enrollment. This sex-specific development of planning was clearly separated from overall intelligence: young boys showed a steeper increase in raw intelligence scores than girls, whereas in the older groups scores developed similarly. The observed sex differences in planning development are evident even within a narrow time window of twelve months and may relate to differences in maturational trajectories for girls and boys in dorsolateral prefrontal cortex.

## Introduction

In contrast to adults' everyday life, the world of children is full of new challenges that cannot be resolved based on previously acquired behavioral routines, but have to be mastered by means of the mental generation and evaluation of behavioral alternatives and their consequences. Previous research has indicated that this ability – to plan ahead – undergoes substantial developmental change especially until the age of six years [1]–[3], hereafter continuing until early adulthood [4]. In an obvious parallel, children in many industrialized societies around the world enter school at this age. Apparently, most children must have reached an adequate level of cognitive and social abilities by this time, making them ready for school enrollment. As mental planning is recognized as essential for academic achievements [5], the observed emergence of planning ability during preschool development thus might pave the way to school aptitude.

**Figure 1:**
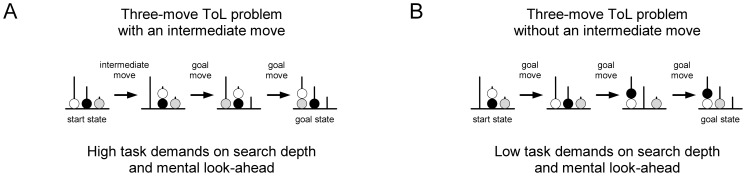
Experimental manipulation of planning demands. Optimal solution of three-move ToL problems either (A) require to mentally look-ahead or (B) can be achieved by placing the balls one after another into their goal position. In the example three-move problem demanding look-ahead, neither the white ball nor the gray ball can be placed directly into its goal position. There are three alternatives for initially moving one of them but only depositing the white ball onto the black ball leads to an optimal solution. Identifying and invalidating the other alternatives clearly requires mentally looking ahead, whereas problems without an intermediate move can be solved in a straight-forward manner, placing each ball in its goal position directly [2].

Recently, the developmental trajectories of basic executive functions were pinpointed in preschool children (e.g. [6]). Whereas inhibition and pure short-term storage are early-developing and basically present in children at the age of 4 and even below, working memory and cognitive flexibility (i.e. set shifting/task switching) evolve later [7]. Whereas these executive functions have been described as developing gradually during childhood, Kaller et al. [2] have recently observed a seemingly qualitative change in planning ability during preschool age using the Tower of London task (ToL). Groups of four- and five-year-old children mastered three-move problems equally well when each ball could be placed into its goal position directly, thus not necessitating mental look-ahead. In sharp contrast, four year olds’ accuracy declined specifically in three-move problems that required planning in terms of mentally looking ahead [2]. This is the case when at least one of the balls cannot be placed directly into its goal position but has to be placed into an intermediate position temporarily, in order to free the way for another ball's goal placement. For efficient performance beyond trial and error, the child has to realize this situation and, accordingly, has to set subgoals (e.g. releasing a blocked position) and to find an optimal way to attain them (Fig. 1). Thus, coping with these problems demands the detection of interdependencies between individual moves that potentially block or enable each other, and therefore clearly necessitates mental look-ahead. Finally, this results in a hierarchical organization of action, including the overall goal(s) as well as necessary temporary subgoals that serve to accomplish them. During individual development the emergence of this planning ability may clearly denote that a child no longer depends on stimulus- or event-dependent chaining of single acts to longer sequences, but has become able to use a more flexible planning mechanism [3].

Here we sought to substantiate this suggestion of a qualitative step forward in individual development by longitudinally tracking the planning abilities of four- and five-year-old children at a retest-interval of 12 months, thus covering the presumably critical time window. To test whether developmental change of planning ability is specific to mental look-ahead or alternatively is related to general cognitive improvements, we also assessed fluid intelligence at the two time points.

In this specific age range, cross-sectional pilot data show that the association of emerging planning ability and maturation of gray matter in the dorsolateral prefrontal cortex is moderated by age and sex (Kaller et al, unpublished data/forthcoming [41]). As is evident from other neuroanatomical studies, girls and boys show different developmental trajectories in gray matter maturation, with girls reaching their peak gray matter volume earlier than boys [8], [9]. Considerable sex differences in frontal gray matter seem to exist already at the age of seven [8] and possibly even earlier. Also, patterns of intrinsic functional connectivity show sexually dimorphic development across childhood [10].

From a cognitive perspective, the notion that men perform better on visuospatial tasks and women on verbal tasks has become common knowledge from adolescent and adult studies [11], [12], [13]. More recent evidence indicated that sex differences occur already in early childhood, favouring boys in visuospatial tasks [14] and girls in multiple measures of language until the age of six [15]. Sex was therefore included as an additional factor in the present analyses to examine likely differences between girls and coeval boys regarding their cognitive development.

## Materials and Methods

### Age Groups

A sample of 62 healthy and unmedicated preschoolers (aged from 50 to 72 months at the first measurement) with normal or corrected-to-normal vision were tested after their parents had given written informed consent. Participants were recruited via newspaper advertisements and word of mouth. The study protocol was approved by the ethics commission of the University of Freiburg (vote number 201/06). Behavioral testing was part of a project including neurophysiological and neuroanatomical measurements. Here, only behavioral data of the planning task and the intelligence test will be reported. The neuroanatomical and neurophysiological data will be analyzed and presented separately.

A total of 16 children dropped out from longitudinal assessments as they refused to perform magnetic resonance imaging (MRI) at the first measurement. Additionally, one boy was excluded due to low performance in the intelligence test (German version of the Coloured Progressive Matrices; [16]). The final sample thus included 45 preschoolers (25 boys, 20 girls) of two age groups: a young group around four years (group 1: n = 22, 12 girls; *M*  =  4;4 years, *SD*  = .19; range  =  4;2 to 4;8 years) and an older group of five year olds (group 2: n = 23, 8 girls; *M*  =  5;4 years, *SD*  = .38, range =  4;9 to 5;9 years). Age did not differ between girls and boys neither in the younger nor in the older group; *t*(20)  =  0.657, *p*  = .519, and *t*(21)  =  1.895, *p*  = .072, respectively.

We also assessed the number of attained school years as well as the highest educational level reached by the children’s mothers and fathers. There were no significant educational differences between parents of the two age groups nor between the girls and boys parents′ in their respective age group (highest *T*  =  1.2; lowest *p*  = .262).

### Planning Task, Setup, and Instructions

Children were tested individually with a computerized three-ball version of the Tower of London (ToL; for overviews, see [17], [18]) task. Start state and goal state were presented in the lower and upper half of the screen, respectively. Children were told to transform the start state into the goal state using a computer mouse while following three rules: (1) only one ball may be moved at a time, (2) a ball cannot be moved while another is on top of it, and (3) three balls may be placed at maximum on the highest peg to the left, two balls on the peg in the middle, and one ball at the peg to the right. To match the goal state, children had to operate on the start state. Movements were executed on a touch screen to avoid potential confounding between planning performance and motor skills in handling a computer mouse. The experimenter stayed in the room for the complete duration of the experiment.

Individual trials were self-initiated by the subject by pressing a button on the screen. After completing the last move required to achieve the goal state the subject received an acoustic feedback to indicate that the problem had been solved. The feedback's valence was always positive, irrespective of solution accuracy, in order to maintain the children's motivation throughout the experiment. Trials exceeding the time-out limit of one minute were automatically aborted (8 % of the trials). If the time limit was exceeded on three consecutive trials, the task was automatically abandoned. Before displaying the next problem, the program acoustically prompted the subject to plan ahead first. After practice in four two-move problems, children’s planning ability was assessed in a total of eight three-move problems.

### Design and Problem Set

Overall, eight 3-move problems had to be solved by the children, two of each problem type as depicted in Table 1. In addition to the experimental manipulation of search depth, the physical appearance of the goal tower configurations was also considered (cf. [19]; see also Table 1). Building on a previous cross-sectional study [2] where we found an age by search depth but no age by goal hierarchy interaction in four- and five-year-old children, the present report is however focused on search depth only. For this purpose, we analyzed search depth for problems with an identical goal hierarchy pattern, that is in partially ambiguous goal tower configurations, only (problem type P2 and P3 in Table 1). As obvious from Fig. 1, search depth concerns whether an intermediate move is needed or not to optimally solve a problem. Problem items and their order of presentation comprised the subset of three-move problems from the standard ToL problem set suggested by Kaller et al. [19]. When retesting the children 12 month later, the same problem set was applied. Task demands on mental look-ahead were systematically varied in a within-subject design with the factor search depth (requiring versus not requiring mental look-ahead) while controlling for the influence of other structural problem parameters. Due to the TOL problem space, the combination of both parameters (search depth and goal hierarchy) results in an unbalanced design. However, to allow for an factorial analysis, the composition of the two structural problem parameters can be transformed into a 2×2 design by nesting the relative ambiguity of subgoal ordering, i.e. goal hierarchy, under the levels of search depth (for details see [20]). For reasons of completeness, the results of this way of analysis are provided at Unterrainer et al. Supporting Information.doc.

**Table 1:** Experimental design for 3-move problems concerning the four resulting problem types (P1-P4), search depth and its move patterns (0  =  intermediate move; 1  =  goal move), and goal hierarchy.

Problem Type	Search Depth	Move Pattern	Goal Hierarchy
P1	Low (no intermediate move)	111	Unambiguous
P2		111	Partially ambiguous
P3	High (one intermediate move)	011	Partially ambiguous
P4		011	Completely ambiguous

### General Intelligence

Intelligence was assessed with the Coloured Progressive Matrices Test (3^rd^ edition, [16]) which measures non-verbal “fluid” intelligence (reasoning, problem solving, judgment, and concept formation), using visual pattern matching and analogy problems pictured in non-representational designs. The CPM comprises 36 items (three sets a 12 item) and the duration of the test was about 20 to 30 minutes.

## Results

Analysis of solution accuracy concerned whether a problem was solved in the minimum number of three moves.

### Planning performance

Individual performance data were entered into a 2×2×2×2 repeated-measurements ANOVA with time point (1^st^ and 2^nd^ measurement after one year; T1 and T2) and search depth as within-subject factors and age group and sex as between-subject factors. Results revealed significant main effects for time point, *F*(1,41)  =  11.92, *p <*.001, η_p_ *^2^*  = .225, search depth, *F*(1,41)  =  44.97, *p <*.001, η_p_ *^2^*  = .523, and age group, *F*(1,41)  =  8.41, *p  = *.006, η_p_ *^2^*  = .170. Children increased performance within the re-test interval of one year, problems with intermediate moves were more difficult to solve, and older children outperformed younger ones. No main effect was observed for sex, *F*(1,41)  = .003, *p  = *.956, η_p_ *^2^*  = .001. There was a significant interaction between time point and age group, *F*(1,41)  =  4.30, *p  = *.044, η_p_ *^2^*  = .095, indicating that younger children performed much worse than older children specifically in the first measurement but could catch up considerably one year later. Analysis of performance separately for each time point revealed that older children outperformed younger children significantly in the first (t(43)  =  3.03, p  = .004), but not in the second measurement (t(43)  =  1,15, p  = .255).

**Figure 2:**
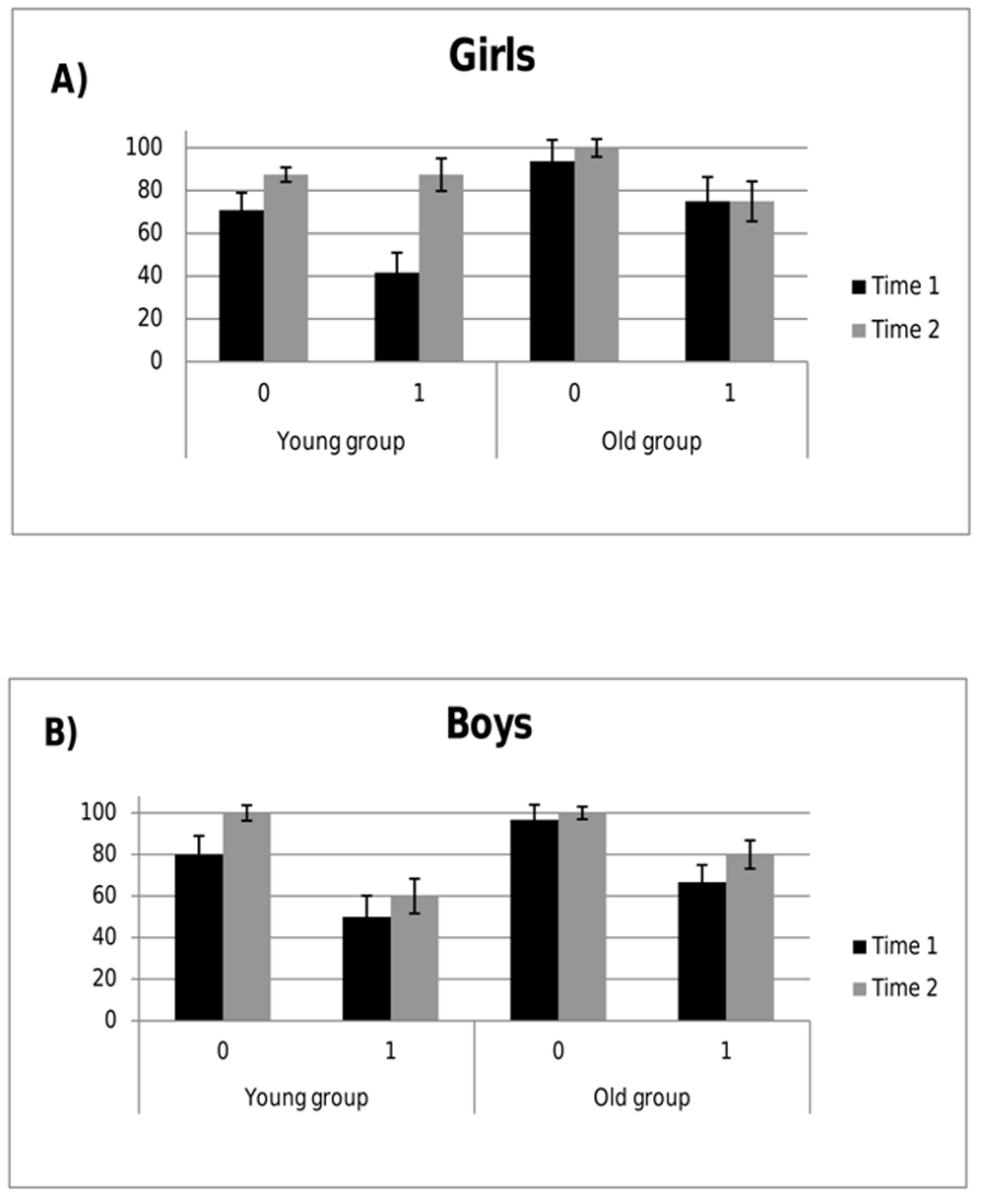
Planning performance as percentage of correctly solved trials in (A) girls and (B) boys in ToL problems not demanding ( = 0) or demanding ( = 1) mental look-ahead. Data are separately plotted for the young and the old group at the time points of the first (Time 1) and the re-test measurement after 12 months (Time 2).

The most striking finding was observed in terms of a quadruple interaction of time point, search depth, age group, and sex, *F*(1,41)  =  4.83, *p  = *.034, η_p_ *^2^*  = .105. As obvious from Figure 2, young boys at age four showed only moderate performance increases in solving intermediate moves one year later, whereas the coeval girls more than doubled their performance and reached a performance level comparable to that of six-year-old boys and girls at the second measurement (Fig. 2). Post hoc analyses confirmed that in the younger children group a highly significant increase for solving intermediate moves could be observed after one year in girls (t(11)  =  –6.17, p <.001), but not for boys (t(9)  =  –.61, p  = .555). In order to preclude that this difference was driven by sample differences between younger and older children, we compared the performance of solving intermediate moves of 5-year-olds at T2 from the younger sample with the 5-year-olds at T1 from the older sample. We could neither find a significant difference for girls (t(18)  =  1.13, p  = .275) nor for boys (t(23)  =  –.474, p  = .640).

### Intelligence

**Figure 3:**
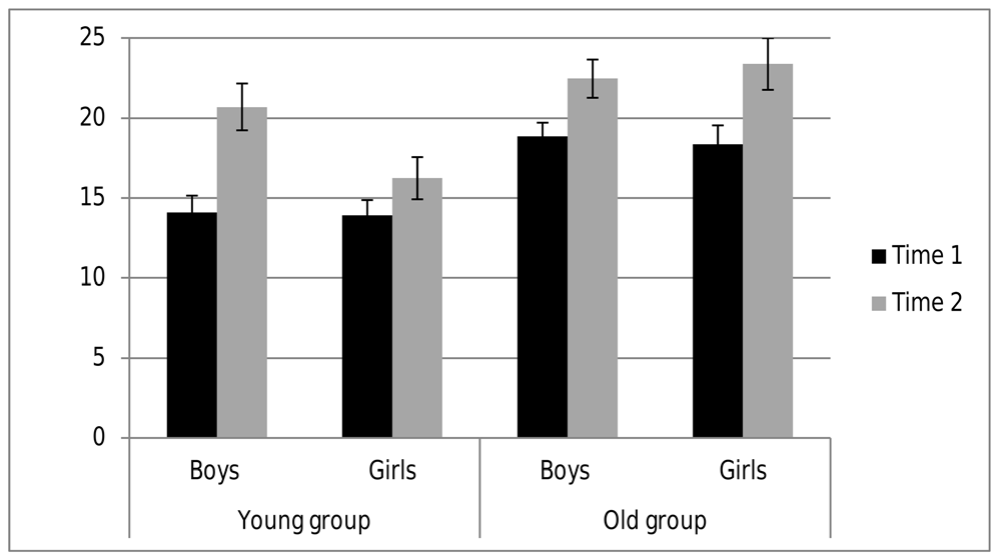
Number of correctly solved items in the intelligence test (CPM) of boys and girls separately plotted for the young and the old group at the time points of the first (Time 1) and the re-test measurement after 12 months (Time 2).

To contrast the development of planning with general intellectual functioning, a 2×2×2 repeated-measurements ANOVA with time point (1^st^ and 2^nd^ measurement after one year; T1 and T2) as within-subject factors and age group and sex as between-subject factors was computed for the number of correctly solved items in the CPM. Results revealed significant main effects for time point, *F*(1,41)  =  47.70, *p <*.001, η_p_ *^2^*  = .538, and age group, *F*(1,41)  =  18.59, *p <*.001, η_p_ *^2^*  = .312. Thus, children gained higher intelligence values twelve months later at T2 and older children significantly outperformed the younger ones. No main effect for sex was found, *F*(1,41)  =  1.02, *p  = *.321, η_p_ *^2^*  = .024. A triple interaction of time point, age group, and sex, *F*(1,41)  =  4.98, *p  = *.031, η_p_ *^2^*  = .108, revealed an inverse pattern to that observed for planning performance: Young boys at age four demonstrated a strong increase in solving items of the intelligence test one year later, whereas young girls only modestly improved at the second measurement (Fig. 3). Post hoc analyses in the younger group revealed highly significant improvements from T1 to T2 in boys (t(9)  =  –6.31, p <.001), but not in girls (t(11)  =  –1.42, p  = .183). Whereas in the older group, both girls (t(7)  =  –4.24, p  = .004) and boys (t(14)  =  –4.03, p  = .001) increased their general intellectual functioning within one year.

## Discussion

The preschool age between four and six years is a time period of pervasive changes in children's thinking [6], as evident in the rapid development of executive functions. The ability to plan ahead, as investigated in the present study, constitutes a prototypical example of *complex* executive functions and is particularly important for successful organization of behavior in situations beyond routine [21].

In basic executive functions, sex differences have been reported earlier for the transition from preschool age to school age, however the extant literature is rather inconsistent: Some studies observed no sex differences (e.g. [22], [23]) whereas in other studies, girls outperformed boys (e.g. [24], [25]).

By employing a longitudinal design in preschool children, here we showed for the first time that sex differences regarding complex executive functions like planning ability follow a specific developmental trajectory. When analyzing the present data for potential differences between girls and boys, we observed a tremendous increase in planning performance that was accomplished in girls one year earlier compared to boys, namely from age four to five. That is, the boys’ development of planning ability lagged behind one year, showing an upswing from age five to six at which boys finally reached the girls' level. Importantly, this sex-specific planning progress was observed only for problems that required mental look-ahead. As obvious from Fig. 2A/B at age five, boys showed a lower performance than girls in both age groups. Presumably due to the small sample sizes, this difference did not attain significance in the old group at time 1, whereas in the young group (time 2) and in the comparison of all five year olds, girls reached significantly higher performances than boys in problems with mental look ahead (*T* =  –2.9; *p* = .008 and *T* =  –2.7; *p* = .009, respectively).

Additionally, we analyzed preplanning times to reveal more information about potential differences in the strategies used. Interestingly, we only found main effects for search depth (problems with mental look ahead had longer planning times a phenomenon repeatedly found in adults), time (children got faster one year later), and age group (younger children needed longer than older). No significant interactions were observed for sex, search depth, age group, and time.

In problems that did not demand look-ahead, even the youngest children reached a rate of 75 % correct solutions, clearly demonstrating that they could follow the task instructions and deal with the working memory load imposed by three-move problems (cf. [2]). Yet, four-year olds (young group, first time point), again irrespective of their sex, only sporadically solved problems that required mental look-ahead, yielding an average accuracy of less than 50 percent. However, at the age of six – when starting their academic career – most of the boys and girls were able to solve these problems that required planning in terms of mental look-ahead and consideration of potential interdependencies between moves.

Noticeably, the observed advantage of young girls for the mental look-ahead ability cannot be explained by general increased intellectual functioning, as coeval young boys showed higher growth in fluid intelligence tasks within this time period. This was rather unexpected since in earlier studies on adults fluid intelligence and planning ability showed modest, but significant positive associations between r  = .34 and r  = .40 [26], [27]. Nevertheless, in these studies planning and visuospatial reasoning were considered as two different cognitive constructs. This consideration is fostered by the fact that fluid intelligence tests using matrices tasks show higher correlations to typical visuospatial tests than the Tower of London-task [27], [28]. Higher visuospatial ability in young boys [14] could thus explain why boys in our study outperformed girls in the CPM.

A potential explanation for the different developmental trajectories in planning ability of boys and girls might be differences in brain development between the ages four and five. Most studies considering sex differences in brain maturation during childhood report a protracted development for males compared to females e.g. [8], [9], [29]). However, these data have to be treated with some caution because particularly those studies starting already at age three and four, though using a longitudinal design, had very small samples in this early age window [30], [31]. Especially for functional neuroimaging studies in early childhood, only few studies used longitudinal design [32].

Another explanation for the differences in CPM and TOL performance between boys and girls may be that the two tasks rely on dissociated neural bases in prefrontal cortex. For adults [33], and already for young children (e.g. [34]), the role of rostrolateral prefrontal cortex especially in more complex 2-relational matrix problems was repeatedly emphasized in neuroimaging studies. In contrast, for the TOL, a predominant role is assigned to dorsolateral prefrontal cortex (e.g. [29], [35]). Given findings that within prefrontal cortex the dorsolateral part matures latest [36], [37], the recruitment of different brain regions of both cognitive domains in combination with differences in brain maturation could serve as an explanation why at the age of four to five girls attain a higher planning skill level whereas at the same age boys perform superiorly in matrix tasks.

### Limitations of the study

The relatively small sample size clearly impacts the conclusions drawn from the present data. This was due to the fact that the behavioral testing was one part of a bigger project and a several children dropped out after the first time point. Nevertheless, the within-subject design as used for this longitudinal study still provides statistical and conceptual advantages that excel cross-sectional designs with greater sample sizes. Time constraints for the behavioral testing hampered additional examinations which may have addressed potential strategy changes in children's performance [38] or provided additional measures to more closely examine sex differences in relation to visuospatial and verbal domains, or potential complementary dimensions such as inhibition or self control [39], [40]. As a consequence, one has to admit more time for behavioral assessment to reduce these constraints in future studies. But the basic results of our present data confirmed previous studies examining the impact of problems that required planning in terms of mentally looking ahead [2] and thus the implications are qualified.

Taken together, mental planning is regarded essential for academic achievement [5]. With our present behavioral results we show that the core ability of planning – namely to mentally look ahead – leaps forward between the age of four and six and is well established when children are ready for school enrollment. This applies for both sexes, but girls attain this mental step one year earlier than boys. As neuroanatomical studies reported sex differences in brain development to occur already in this age range, these may provide an important source for variation in cognitive development. However, the direct link of the observed asynchrony in cognitive developments between girls and boys to an underlying differential course of brain maturation will have to be established in further analyses.

## Supporting Information

File S1 Supporting Information
(DOC)

## References

[1] Klahr D, Robinson M (1981) Formal assessment of planning and problem solving in preschool children. Cogn Psychol 13: 113–48.

[2] Kaller CP, Rahm B, Spreer J, Mader I, Unterrainer JM (2008) Thinking around the corner: the development of planning abilities. Brain Cogn 67: 360–70. 18440114 10.1016/j.bandc.2008.02.003

[3] McCormack T, Atance CM (2011) Planning in young children: A review and synthesis. Dev Rev 31: 1–31.

[4] Albert D, Steinberg L (2011) Age differences in strategic planning as indexed by the Tower of London. Child Dev 82: 1501–1517. 21679178 10.1111/j.1467-8624.2011.01613.x

[5] Meltzer LJ (2007) Executive function in education: From theory to practice. New York: Guilford. 337 p.

[6] Garon N, Bryson SE, Smith IM (2008) Executive function in preschoolers: A review using an integrative framework. Psychol Bull 134: 31–60. 18193994 10.1037/0033-2909.134.1.31

[7] Davidson MC, Amso D, Anderson LC, Diamond A (2006) Development of cognitive control and executive functions from 4 to 13 years: evidence from manipulations of memory, inhibition, and task switching. Neuropsychologia 44: 2037–78. 16580701 10.1016/j.neuropsychologia.2006.02.006 PMC1513793

[8] Lenroot RK, Gogtay N, Greenstein DK, Wells EM, Wallace GL, et al (2007) Sexual dimorphism of brain developmental trajectories during childhood and adolescence. Neuroimage 15: 1065–73. 10.1016/j.neuroimage.2007.03.053 PMC2040300 17513132

[9] Raznahan A, Lerch JP, Lee N, Greenstein D, Wallace GL, et al (2011) Patterns of coordinated anatomical change in human cortical development: a longitudinal neuroimaging study of maturational coupling. Neuron 8: 873–84. 10.1016/j.neuron.2011.09.028 PMC4870892 22153381

[10] Zielinski BA, Gennatas ED, Zhou J, Seeley WW (2010) Network-level structural covariance in the developing brain. Proc Natl Acad Sci U S A 19: 18191–6. 10.1073/pnas.1003109107 PMC2964249 20921389

[11] Maccoby E, Jacklin C (1974) The Psychology of Sex Differences. Standford CA: Standford University Press.

[12] Halpern DF (1997) Sex differences in intelligence. Implications for education. Am Psychol 52: 1091–102. 9329293 10.1037//0003-066x.52.10.1091

[13] Kimura D (1992) Sex differences in the brain. Sci Am 267: 118–25. 1298222 10.1038/scientificamerican0992-118

[14] Hahn N, Jansen P, Heil M (2010) Preschoolers' mental rotation: sex differences in hemispheric asymmetry. J Cogn Neurosci 22(6): 1244–50. 19366287 10.1162/jocn.2009.21236

[15] Bornstein MH, Hahn C-S, Haynes OM (2004) Specific and general language performance across early childhood: Stability and gender considerations. First Lang 24(3): 267–305.

[16] Bulheller S, Häcker HO (2002) Coloured Progressive Matrices (CPM). Deutsche Bearbeitung und Normierung nach J. C. Raven. Frankfurt: Pearson Assessment.

[17] Berg WK, Byrd D (2002) The Tower of London spatial problemsolving task: Enhancing clinical and research implementation. J Clin Exp Neuropsychol 24: 586–604. 12187443 10.1076/jcen.24.5.586.1006

[18] Berg WK, Byrd DL, McNamara JPH, Case K (2010) Deconstructing the tower: Parameters and predictors of problem difficulty on the Tower of London task. Brain Cogn 72: 472–482. 20167413 10.1016/j.bandc.2010.01.002

[19] Kaller CP, Rahm B, Köstering L, Unterrainer JM (2011a) Reviewing the impact of problem structure on planning: a software tool for analyzing tower tasks. Behav Brain Res 216: 1–8. 20723568 10.1016/j.bbr.2010.07.029

[20] McKinlay A, Kaller CP, Grace RC, Dalrymple-Alford JC, Anderson T, et al (2008) Planning in Parkinson's Disease: A matter of problem structure? Neuropsychologia 46(1): 384–9. 17928014 10.1016/j.neuropsychologia.2007.08.018

[21] Ward G, Morris R G (2005) Introduction to the psychology of planning. In Morris R, Ward G, editors. The cognitive psychology of planning. Hove, UK: Psychology Press. pp. 1–34.

[22] Welsh MC, Pennington BF, Groisser DB (1991) A normative developmental study of executive function: A window on prefrontal function in children. Dev Neuropsychol 7: 131–149.

[23] Hughes C, Ensor R, Wilson A, Graham A (2010) Tracking executive function across the transition to school: a latent variable approach. Dev Neuropsychol 35: 20–36. 20390590 10.1080/87565640903325691

[24] Klenberg L, Korkman M, Lahti-Nuuttila P (2001) Differential development of attention and executive functions in 3- to 12-year-old Finnish children. Dev Neuropsychol 20: 407–28. 11827096 10.1207/S15326942DN2001_6

[25] Böhm B, Smedler AC, Forssberg H (2004) Impulse control, working memory and other executive functions in preterm children when starting school. Acta Paediatr 93: 1363–71. 15499959 10.1080/08035250410021379

[26] Gilhooly KJ, Wynn V, Phillips LH, Della Sala S (2002) Visuo-spatial and verbal working memory in the five-disc Tower of London task: An individual differences approach. Think Reason 8: 165–178.

[27] Unterrainer JM, Rahm B, Kaller CP, Leonhart R, Quiske K, et al (2004) Planning abilities and the Tower of London: is this task measuring a discrete cognitive function? J Clin Exp Neuropsychol 26(6): 846–856. 15370380 10.1080/13803390490509574

[28] Cheetham JM, Rahm B, Kaller CP, Unterrainer JM (2012) Visuospatial over verbal demands in predicting Tower of London planning tasks. Br J Psychol 103(1): 98–116. 22229777 10.1111/j.2044-8295.2011.02049.x

[29] Kaller CP, Rahm B, Spreer J, Weiller C, Unterrainer JM (2011b) Dissociable Contributions of Left and Right Dorsolateral Prefrontal Cortex in Planning. Cereb Cortex 21: 307–17. 20522540 10.1093/cercor/bhq096

[30] Sowell ER, Thompson PM, Leonard CM, Welcome SE, Kan E, et al (2004) Longitudinal mapping of cortical thickness and brain growth in normal children. J Neurosci 24: 223–8231. 10.1523/JNEUROSCI.1798-04.2004 PMC6729679 15385605

[31] Giedd JN, Blumenthal J, Jeffries NO, Castellanos FX, Liu H, et al (1999) Brain development during childhood and adolescence: a longitudinal MRI study. Nat Neurosci 2: 861–863. 10491603 10.1038/13158

[32] Moriguchi Y, Hiraki K (2011) Longitudinal development of prefrontal function during early childhood. Dev Cogn Neurosci 1(2): 153–162. 22436437 10.1016/j.dcn.2010.12.004 PMC6987577

[33] Christoff K, Prabhakaran V, Dorfman J, Zhao Z, Kroger JK, Holyoak KJ, Gabrieli JD (2001) Rostrolateral prefrontal cor­tex involvement in relational integra­tion during reasoning. Neuroimage 14: 1136–1149. 11697945 10.1006/nimg.2001.0922

[34] Ferrer E, O'Hare ED, Bunge SA (2009) Fluid reasoning and the developing brain. Front Neurosci 1 3(1): 46–51. 10.3389/neuro.01.003.2009 PMC2858618 19753096

[35] Unterrainer JM, Owen AM (2006) Planning and Problem Solving: from Neuropsychology to Functional Neuroimaging. J Physiol Paris 99: 308–317. 16750617 10.1016/j.jphysparis.2006.03.014

[36] Gogtay N, Giedd JN, Lusk L, Hayashi KM, Greenstein D, et al (2004) Dynamic mapping of human cortical development during childhood through early adulthood. Proc Natl Acad Sci U S A 101: 8174–8179. 15148381 10.1073/pnas.0402680101 PMC419576

[37] Badre D, D'Esposito M (2009) Is the rostro-caudal axis of the frontal lobe hierarchical? Nat Rev Neurosci 10 (9): 659–669. 10.1038/nrn2667 PMC3258028 19672274

[38] Lemaire P, Siegler RS (1995) Four aspects of strategic change: contributions to children's learning of multiplication. J Exp Psychol Gen 124(1): 83–97. 7897342 10.1037//0096-3445.124.1.83

[39] Silverman I (2003) Gender differences in resistance to temptation: theories and evidence. Dev Rev 23: 219–259.

[40] Stevenson JC, Williams DC (2000) Parental investment, self-control, and sex differences in the expression of ADHD. Hum Nat 11: : 405–422. 10.1007/s12110-000-1010-6 26193660

[41] KallerCP, SpreerJ, RahmB, GlaucheV, RuhNet al.. (in press) Exploring the neuroanatomy of planning abilities in early childhood with voxel-based morphometry. Poster presented at the 2010 Annual Meeting of the Organization for Human Brain Mapping, Jun 06-10th 2010, Barcelona, Spain.

