# Supplementary material for: Planning Steps Forward in Development: In Girls Earlier than in Boys
Source: PLoS One. 2013 Nov 27;8(11):e80772. doi: 10.1371/journal.pone.0080772 (PMC3842368; doi:10.1371/journal.pone.0080772)
Supplement: File S1 — Supporting Information (DOC) [file pone.0080772.s001.doc]

Supporting Information

As we focused on search depth in the main analyses, we here provide further results regarding the factorial analyses of both search depth and goal hierarchy. As already demonstrated [20] the two structural problem parameters can be transformed into a 2×2 design by nesting the relative ambiguity of subgoal ordering, i.e. goal hierarchy, under the levels of search depth.

Individual performance data were mean-aggregated across trials and entered into a 2x2x2x2x2 repeated-measurements ANOVA with time (1^st^ and 2^nd^ test session), goal hierarchy and search depth as within-subject factors and age group and sex as between-subject factors. Results revealed significant main effects for time, *F*(1,41) = 13.10, *p =* .001, η_p_*^2^* = .242, search depth, *F*(1,41) = 52.46, *p <* .001, η_p_*^2^* = .561, and age group, *F*(1,41) = 11.94, *p =* .001, η_p_*^2^* = .226. Children increased performance within one year, problems with intermediate moves were more difficult to solve, and older children outperformed younger ones. There were no main effects for goal hierarchy or sex. As in Kaller et al. [2], there was a significant interaction between search depth and age group, *F*(1,41) = 4.30, *p =* .044, η_p_*^2^* = .095, indicating that younger children performed low specifically in problems requiring mental look-ahead. In addition, we could also find a two-way interaction of goal hierarchy with age groups, *F*(1,41) = 6.57, *p =* .014, η_p_*^2^* = .138, that revealed an increase of correctly solved problems with high ambiguity in the older and a decrease in the younger group in relation to goal configurations with low ambiguity. There was also a triple-interaction of search depth, goal hierarchy and sex, *F*(1,41) = 4.54, *p =* .039, η_p_*^2^* = .100, showing that in boys only search depth affected planning accuracy independently of the goal configuration, whereas girls showed performance differences for problems with and without intermediate moves when the goal configuration had a low ambiguity, with a more stable search depth performance in ambiguous problems.

The most interesting findings concerning the development were those including the factor “time”, that reflects the one year retest-interval, such as the triple interaction of time, age groups and sex, *F*(1,41) = 5.07, *p =* .030, η_p_*^2^* = .110: Both younger and older boys showed an increase of performance across time, whereas in girls only the younger group showed enhanced performance after 12 months, since the older group showed a very high performance level already at the first time point. This general planning phenomenon was more specifically expressed in the interaction of time, search depth and sex, *F*(1,41) = 4.21, *p =* .047, η_p_*^2^* = .093, reflecting that in boys problems requiring mental look-ahead were significantly more difficult at both time points, whereas in girls this was evident at time point 1 only, but not one year later. Until then, the girls' performance in problems requiring mental look-ahead had reached approximately the same level as in those not requiring look-ahead. Finally, the most interesting and highly significant findings could be observed in a quadruple interaction of time, search depths, age groups, and sex *F*(1,41) = 9.79, *p =* .003, η_p_*^2^* = .193. Here, the effect described above could even be more specified with age groups: young boys at age four show the same problems in solving intermediate moves one year later, whereas the coeval girls already reached performance level at the second measurement as did older boys and girls at age six (Fig 2).

Thus, this effect fully corresponds to the analyses reported in the main manuscript, which compared only the subset of problems requiring vs. not requiring mental look-ahead at a partially ambiguous goal hierarchy.
